# Short-term outcome following postoperative enhanced recovery implementation in patients with perforated peptic ulcer

**DOI:** 10.1186/s40001-025-02432-7

**Published:** 2025-04-04

**Authors:** Amna A. Desoky, Mahmoud T. Ayoub, Neama M. Mostafa, Eman M. Hashem, Mona A. Mohammed

**Affiliations:** 1 Department of Medical-Surgical Nursing, Faculty of Nursing, Assiut University, Assiut, 71515 Egypt; 2 Department of General Surgery, Faculty of Medicine, Assiut University, Assiut, Egypt; 3 Department of Medical Physiology, Faculty of Medicine, Assiut University, Assiut, Egypt

**Keywords:** Outcome, Perforated peptic ulcer, Postoperative enhanced recovery

## Abstract

**Objectives:**

To evaluate the short-term outcome following postoperative enhanced recovery implementation in patients with perforated peptic ulcer.

**Methods:**

Quasi-experimental research design was utilized. Thirty patients received postoperative enhanced recovery after open surgical repair of perforated peptic ulcer compared with control group who received routine care. Patient assessment sheet and gastrointestinal quality of life index were the tools used for data collection.

**Results:**

The mean age was 40.43 ± 8.39 years for the study group and 39.53 ± 8.08 for the control group (56.7%, 70%), respectively, were males. The study group demonstrated early first bowel movement, flatus and stool passage (8.1 ± 1.16 (h), 12.6 ± 2.46 (h), and 2.47 ± 0.82 (days)), respectively, compared to control group (10 ± 1.11, 15.1 ± 2.04, and 3.57 ± 0.82). A significant reduction (6.93 ± 1.29 vs. 12.3 ± 4.96 (days)) and (30% vs. 60%) in hospital length of stay and postoperative complications among study group compared to control group (*P* < 0.01). The mean scores (56.17 ± 13.78 and 72.6 ± 11.89 vs. 34.33 ± 8.91and 53.43 ± 16.14) of gastrointestinal quality of life index were significantly better in study group (*P* < 0.05).

**Conclusion:**

Improved gastrointestinal functional recovery, reduced postoperative complications, and improved quality of life, all were a result of implementing postoperative enhanced recovery among patients with perforated peptic ulcer.

*Trial Registration* Number (TRN) -URL: https://www.clinicaltrials.gov. Unique identifier: NCT06570018 Date of registration July August 22, 2024

## Background

Perforated peptic ulcer (PPU) is a serious complication of peptic ulcer disease (PUD) in which there is a hole in the stomach or duodenal wall leading to leakage of gastric acid bile, and gas into the peritoneal cavity. The incidence of PUD, either gastric or duodenal, has decreased during the last few decades with the discovery of the role of *Helicobacter pylori* (*H. pylori*). However, the incidence of peptic ulcer complications has not decreased in the same manner [1] Bleeding and perforation are the most severe complications of PUD. Due to progress in endoscopic and interventional radiological techniques, bleeding is mostly considered a medical emergency, and outcomes have improved [2, 3].

Perforation remains a surgical emergency, incidence of PPU in the UK is about 12.17 per 100,000 person years. Surgical repair with closure of the perforation, with or without an omental pedicle, is the preferred treatment for PPU. This repair can be achieved through either open repair or laparoscopy which can be associated with a significant postoperative morbidity or mortality [4]. Also, PPU has an extensive impact on a patient’s health status. Several studies have found that people with PPU have lower QOL than the general population and that improving QOL is crucial in the management of PPU [5].

Enhanced recovery after surgery (ERAS) programs represents evidence-based protocols for surgical management. Such protocols include pre-, intra- and postoperative principles to minimize surgical stress and accelerate patient recovery. Successful implementation depends on adoption of multiple ERAS principles and collaboration from all members of the surgical team [6]. In emergency general surgery, enhanced perioperative care remains a “grey area” with little evidence available and a great debate although advocation for the promising results of some studies [7, 8].

### Study objectives and hypothesis

This study aimed to evaluate the short-term outcome following postoperative enhanced recovery implementation in patients with perforated peptic ulcer. In the study setting, it was noted that postoperative recovery of those patients is often very long, painful, expensive, and a highly variable experience. Consequently, it was assumed that implementing postoperative enhanced recovery will find a great utility on this issue.

## Method

### Research design

Quasi-experimental (study–control) research design was utilized to conduct this study. Participants were allocated according to the timeline for ERAS implementation. The first 30 patients, treated between October 2023 and February 2024, served as the control group, receiving conventional care. The subsequent 30 patients, managed from March to July 2024, constituted the study group that received the intervention. To enhance comparability, propensity score matching was used to adjust for differences between groups.

### Setting

The study was conducted in the general surgery department at Assiut University Hospital.

### Participants

The sample size was determined statistically by power analysis. Calculation was done considering the following: Type I error with significant level (α) = 0.5, Type II error by power test (1-B) = 80% and found the minimum sample size was 27 patients for each group. Although the minimum number of 54 patients was required by power analysis, the researcher had obtained 60 patients in this study because non-response rate was expected to be lost from the subjects.

A purposive sample of adult patients that their age ranged from 18 to 65 years, diagnosed with perforated peptic ulcer, ulcer size less than 10 mm, and underwent emergent surgical intervention were eligible for the study. Patients with a malignant ulcer, other GIT diseases (Crohn’s disease and ulcerative colitis), pregnancy, psychiatric or neurological illness, and who refused to participate in the study were excluded. The study group received postoperative enhanced recovery elements while the control group received routine care.

### Study instruments

#### Patient assessment sheet

It was developed by researchers based on literatures review to assess demographic. Clinical data included risk factors, causes of perforated peptic ulcer, comorbid condition, site of perforation, size of ulcer, duration of preoperative symptoms, and preoperative lab investigations as baseline assessment. Postoperative physiological parameters pertaining gastrointestinal functional recovery as postoperative first bowel movement, first flatus, and defecation formulated as a primary outcome. Thirty days postoperative complications that anticipated to occur within 30 days postoperative as pneumonia, admission to critical care, abdominal collection, intra-abdominal abscess, suture leakage, omental patch leakage, septic shock, prolonged ileus, surgical site infection, urinary tract infection, deep venous thrombosis (DVT), and reoperation as well as length of hospital stay were included as primary outcomes too.

#### Gastrointestinal quality of life index (GIQLI)

Quality of life was evaluated as secondary outcome by using gastrointestinal quality of life index (GIQLI). It is a 36-item scale divided into five domains; gastrointestinal (GIT) symptoms (10 items), physical (6 items), emotion (6 items), social (2 items) and disease specific (8 items) Cronbach's alpha was 0.89 [9]. Each item is based on a 0–4 Likert scale with 0 being least desirable to 4 being the most desirable option. The total scores represent Gastrointestinal Quality of Life Index. It ranges from 0 to 144 with higher scores indicating a better quality of life.

### Data collection

The process of data collection started from the beginning of October 2023 to the mid of July 2024. Each patient either in study or control group arrived to the surgical team after the office hours with perforated peptic ulcer, within two hours; the surgical intervention would be initiated. During the two hours, the researcher assessed demographic and clinical data. During this phase the role of researchers was just observing the tasks performed concerning pre- and intra-operative care. Both the study and control groups received early intravenous broad-spectrum antibiotics targeting gram-negative, positive, and anaerobe bacteria. The treatment protocol consisted of a third-generation cephalosporin (Ceftriaxone and Sulbactam, 1.5 g IV) combined with Metronidazole 500 mg intravenously administered preoperatively and continued after surgery. The surgical interventions in both groups were performed by surgeons with equivalent experience levels, including one gastrointestinal (GIT) specialist and one senior resident. The surgeons were blinded during the operation but were informed about patient allocation after surgery.

Postoperative care for both groups was similar while the difference was in the term of ERAS for the study group and routine hospital care for the control group. According to routine care for the control group, parenteral fluid intake was controlled on the day after the surgery up to the third day. The dietary plan after surgery involved a gradual progression, starting with no oral intake for 3 days, followed by 3 days of oral fluids, and then patients were introduced to semi-solid and last to solid food. The surgical drain was removed between the third and fifth day after the operation, while the nasogastric tube was typically discontinued on the second or third day. Patients were encouraged to get out of bed at their own choice.

### Intervention

According to the study group, the researchers collaborated with surgeons, nurses, and anesthesiologists to implement postoperative enhanced recovery protocol listed in Table 1 that adopted the following elements: Early nutrition, early mobilization, non-opioid analgesia, and early removal of abdominal drains and tubes throughout the patients’ postoperative hospitalization up to discharge [10, 11].

**Table 1:** Detailed elements of the ERAS provided for the study group

Day	Postoperative enhanced recovery care
Post op. (day 1)	▪ Gastric content was aspirated via the nasogastric tube by the anesthesiologist at the end of the operation ▪ Nasogastric tube was withdrawn 24 h postoperative in the department
	▪ Nil by mouth, parenteral fluid therapy
	▪ Pain control to reduce insulin resistance and support mobilization
	▪ Nausea and vomiting control
	▪ Use of chewing gums 6 h postoperative
	▪ Early mobilization ▪ Mobilization on bed (6 h postoperative) ▪ Walk the length of the room (evening day of surgery)
Post op. (day 2)	▪ Liquid diet for 2 days. (day 2,3) Support energy supply ▪ Removal of central venous catheter, arterial line, urinary catheter within 24 h
Pot op. (day 3)	▪ Remove drain (s) ▪ The patients started to eat soft food to support ambulation and mobilization
Pot op. (day 4)	▪ Continue to eat soft food to support protein supply
Pot op. (day 5)	– Discharge instructions
Pot op. (day 6)	– Discharge day 6 or more – Discharge criteria ▪ Full mobilization ▪ Ability to tolerate solids ▪ Presence of active bowel sounds ▪ Absence of any postoperative complications

Post op.: postoperative

Postoperative enhanced recovery protocol was initiated by gradual reduction of intravenous “IV” fluids up to 24 h. postoperative, termination of analgesia. Early nutrition was achieved by chewing gum 6 h. postoperative to stimulate return of gut function. Oral liquids started 24 h. postoperative and nasogastric tube removed, if liquids were tolerated, patients then advance to a soft diet on day two postoperative. Patients were instructed that drinking fluids is more important than eating, also small, frequent meals is better for diet tolerance. If any patient showed inability to tolerate oral diet, the intake was withheld instantly. The criteria for intolerance defined as any of the following: > 100 ml of vomiting with > 2 episodes over 24 h, abdominal distension, moderate-to-severe pain (Likert score > 3/5), or 1 episode of diarrhea.

According to postoperative ambulation, patients had an order to be out of bed a minimum of two times per day, as their condition allows. Foley catheter removed 24 h. postoperative. The decision to continue the maintenance antisecretory therapy varied based on ulcer characteristics and risk factors for PUD recurrence. Patients were discharged when they tolerated a solid diet for at least one day and without developing any complications. Before discharge, patients were instructed about healthy lifestyle, wound care, adherence to medication, and follow-up schedule.

### Outcome

Postoperatively, both groups had been evaluated for primary outcome by measuring physiological parameters (gastrointestinal functional recovery), postoperative complications, and length of hospital stay. Quality of life among patients was evaluated twice as secondary outcome before hospital discharge and 30 days postoperative.

### Statistical analysis

The data were tested for normality using the Anderson–Darling test and for homogeneity variances prior to further statistical analysis. Categorical variables were described by number and percent (N, %), where continuous variables described by mean and standard deviation (mean, SD). Chi-square test or Fisher’s exact test was used as appropriate to compare between categorical variables where compare between continuous variables by “independent-samples t-test” or “paired-samples t-test” as appropriate. A two-tailed “*P* < 0.05” was considered statistically significant. Pearson correlation was used to show the association between variables. All analyses were performed with the IBM SPSS 26 software.

## Results

### Baseline data

Table 2 displays the demographic and clinical data of studied patients. The mean age was 40.43 ± 8.39 years for the study group and 39.53 ± 8.08 for the control group. Nearly half (50%, 53.3%) resp. in both study and control groups belong to the age group 30- > 40 years. The highest percentage (56.7%, 70%), respectively, in both groups were males. According to risk factors, it was noted that exposure to stress (53.3%, 46.7%) and previous use of NSAIDs (50%, 66.7%), respectively, were the predominant than others. According to site of perforation, nearly most (90%, 93.3%) of patients in both groups had duodenal ulcer where ulcer size < 5 mm. Majority (60%, 66.7%) of patients in both study and control groups had symptoms for ≥ 24 h.

**Table 2:** Demographic and clinical data of studied patients

Variables	Study (*n* = 30)	Control (*n* = 30)	X^2^/t	*P* value
Age (years) mean ± SD	40.43 ± 8.39	39.53 ± 8.08	0.423	0.674
Age group *n* (%)				
18 > 30 years *n* (%)	3 (10.0)	4 (13.3)	0.363	0.948
30 > 40 years *n* (%)	15 (50.0)	16 (53.3)		
40 > 50 years *n* (%)	7 (23.3)	6 (20.0)		
50–65 years *n* (%)	5 (16.7)	4 (13.3)		
Gender				
Male *n* (%)	17 (56.7)	21 (70.0)	1.148	0.284
Female *n* (%)	13 (43.3)	9 (30.0)		
Predisposing factors for perforated peptic ulcer				
History of peptic ulcer treatment *n* (%)	7 (23.3)	11 (36.7)	1.270	0.260
Smoking *n* (%)	7 (23.3)	14 (46.7)	3.590	0.058
Stress *n* (%)	16 (53.3)	14 (46.7)	0.267	0.606
NSAIDs use *n* (%)	15 (50.0)	20 (66.7)	1.714	0.190
Spicy food *n* (%)	4 (13.3)	9 (30)	2.455	0.117
Current health history				
Co-morbid *n* (%)	4 (13.3)	7 (23.3)	1.002	0.317
Shock on admission *n* (%)	25 (83.3)	27 (90.0)	0.577	0.448
Delayed surgery > 6 h. of admission *n* (%)	16 (53.3)	18 (60.0)	0.271	0.602
Preoperative investigations				
Metabolic acidosis *n* (%)	10 (33.3%)	14 (46.7%)	1.111	0.292
Hemoglobin < 6.0 mmol/l *n* (%)	11 (36.7%)	10 (33.3%)	0.073	0.787
Creatinine > 110/130 µmol/l *n* (%)	6 (20%)	8 (26.7%)	0.373	0.542
Albumin < 550 µmol/l *n* (%)	9 (30%)	10 (33.3%)	0.077	0.781
Site of perforation				
Stomach *n* (%)	3 (10.0)	2 (6.7)	0.218	0.640
Duodenum *n* (%)	27 (90.0)	28 (93.3)		
Size of ulcer				
< 5 mm *n* (%)	25 (83.3)	24 (80.0)	0.111	0.739
≥ 5 mm *n* (%)	5 (16.7)	6 (20.0)		
Duration of symptoms				
≥ 24 h *n* (%)	18 (60.0)	20 (66.7)	4.286	0.788
< 24 h *n* (%)	12 (40.0)	10 (33.3)		
Duration of operation				
< 2 s *n* (%)	19 (63.3)	19 (63.3)	1.048	0.592
2–4 h *n* (%)	10 (33.3)	11 (36.7)		
> 4 h *n* (%)	1 (3.3)	0 (0.0)		

### Primary outcomes

Table 3 reflects a statistically significant difference between the two groups in which the study group demonstrated early gastrointestinal functional recovery. First bowel movement, first flatus and stool passage (8.1 ± 1.16 (h), 12.6 ± 2.46 (h), and 2.47 ± 0.82 (days)), respectively, compared to control group (10 ± 1.11, 15.1 ± 2.04, 3.57 ± 0.82), respectively. As well there was a significant reduction (6.93 ± 1.29 vs. 12.3 ± 4.96 (days)) in hospital length of stay among study group compared to control group.

**Table 3:** Comparison between mean scores of postoperative gastrointestinal functional recovery and length of hospital stay among participants

Gastrointestinal functional recovery and LOS	Study (*n* = 30)	Control (*n* = 30)	X^2^/t	*P* value
First bowel movement (h)				
Min.–Max	6–10	8–12		
Mean ± SD	8.1 ± 1.16	10 ± 1.11	− 6.484	< 0.001**
First flatus passage (h)				
Min.–Max	3–16	6–17		
Mean ± SD	12.6 ± 2.46	15.1 ± 2.04	− 4.287	< 0.001**
First stool passage (days)				
Min.–Max	1–4	2–5		
Mean ± SD	2.47 ± 0.82	3.57 ± 0.82	− 5.207	< 0.001**
Length of hospital stay (days)				
Mean ± SD	6.93 ± 1.29	12.3 ± 4.96	− 5.200	< 0.001**
≤ 5 days *n* (%)	10 (33.3)	0 (0.0)	17.389	< 0.001**
From 6 to 10 days *n* (%)	19 (63.3)	20 (66.7)		
> 10 days *n* (%)	1 (3.3)	10 (33.3)		

**Significant at *P* > 0.01

Table 4 illustrates statistically significant differences between study and control groups regarding postoperative complications that occurred within 30 days. The number of patients who developed prolonged ileus, and surgical site infection and burst significantly decreased in study group than control group (*P* < 0.05), while there were no significant differences regarding rest of complications.

**Table 4:** Incidence of postoperative complications among studied participants

Postoperative complications	Study (*n* = 30)		Control (*n* = 30)		X^2^	*P* value
	No	%	No	%		
Total postoperative complications						
None	21	70.0	12	40.0	18.20	0.000**
One complication	3	10.0	0	0.0		
Two complications	5	16.7	3	10.0		
Three complications	1	3.3	15	50.0		
One by one postoperative complications						
Admission to ICU	3	10.0	7	23.3	1.92	0.166
Septic shock	1	3.3	3	10.0	1.07	0.301
Pneumonia	2	6.7	7	23.3	3.27	0.071
DVT	0	0.0	1	3.3	1.02	0.313
Urinary tract infection	3	10.0	8	26.7	2.78	0.095
Prolonged ileus	2	6.7	9	30.0	5.46	0.020*
Omental patch leakage	1	3.3	1	3.3	0.00	1.000
Intra-abdominal abscess	1	3.3	3	10.0	1.07	0.301
Surgical site infection and burst	2	6.7	11	36.7	7.95	0.005**
Reoperation	1	3.3	2	6.7	0.35	0.554

Chi-square test for qualitative data between the two groups

*Significant level at *P* value < 0.05, **Significant level at *P* value < 0.05

### Secondary outcome

Table 5 clarifies that the study group had a significant improvement in all domains of gastrointestinal quality of life index before hospital discharge and after 30 days postoperative compared to control group (*P* < 0.05).

**Table 5:** Mean score of gastrointestinal quality of life index (GIQLI)

Domains	Before discharge				Thirty days postoperative			
	Study (*n* = 30)	Control (*n* = 30)	t	*P* value	Study (*n* = 30)	Control (*n* = 30)	t	*P* value
Gastrointestinal symptoms								
Min.–Max	1–23	3–21			9–29	5–29		
Mean ± SD	14.07 ± 5.17	8.03 ± 4	5.056	< 0.001**	18.1 ± 3.82	13.7 ± 6.37	3.244	0.002**
Emotion								
Min.–Max	4–15	1–10			8–21	4–19		
Mean ± SD	9.37 ± 2.71	6.03 ± 2.3	5.140	< 0.001**	12.97 ± 2.62	9.53 ± 3.53	4.278	< 0.001**
Physical function								
Min.–Max	4–19	3–10			8–20	3–19		
Mean ± SD	10.2 ± 3.44	6 ± 2.33	5.536	< 0.001**	13.63 ± 2.99	9.5 ± 4.35	4.293	< 0.001**
Social function								
Min.–Max	0–9	1–6			5–12	1–10		
Mean ± SD	6.17 ± 2.05	3.5 ± 1.55	5.681	< 0.001**	8.17 ± 1.76	5.63 ± 2.51	4.521	< 0.001**
Disease-specific items								
Min.–Max	7–33	5–18			9–32	5–24		
Mean ± SD	16.37 ± 5.42	10.77 ± 3.13	4.904	< 0.001**	19.73 ± 4.61	15.07 ± 5.17	3.692	< 0.001**
Gastrointestinal quality of life index (GIQLI)								
Min.–Max	30–94	22–55			46–100	29–93		
Mean ± SD	56.17 ± 13.78	34.33 ± 8.91	7.287	< 0.001**	72.6 ± 11.89	53.43 ± 16.14	5.236	< 0.001**

**Significant at *P* < 0.01

## Discussion

Enhanced recovery after surgery (ERAS) protocols have been extensively studied in elective abdominal surgeries with promising results. However, in the study sitting, the use of these protocols in emergency abdominal surgeries has not been widely investigated. This study aimed to evaluate the effect of the short-term outcome of implementing postoperative enhanced recovery on perforated peptic ulcer.

The results of the present study displayed that many patients in both groups were males. The mean age was 40.43 ± 8.39 years for the study group and 39.53 ± 8.08 for the control group. In the same line a recent study by [12] who retrospectively studied five-hundred patients underwent surgery for perforated peptic ulcer and found that most of studied sample were males, mean age was 46.5 years, and the site of perforation was in the stomach which is contradict to our finding where the duodenal ulcer was the highest than gastric.

According to risk factors, it was necessary to assess risk factors for the development of perforated peptic ulcer. The results of the current study illustrated that exposure to stress and previous use of NSAIDs were the predominant risk factors than others. This could help researchers to address patients’ needs and give health teaching before discharge to prevent complication and recurrence. Given that perforated peptic ulcer is an emergency surgical experience, all patients received educational needs postoperatively despite this education being recommended before surgery and hospital admission.

Ding et al. [13] reported that patients with peptic ulcer will experience different problems after discharge from the hospital postoperatively. This involves complications associated with self-activity, diet and medication. Also, they added that patients need to continue drug maintenance treatment to achieve symptom relief, ulcer healing, and prevent ulcer recurrence.

The findings of the current study revealed favorable results as the gastrointestinal functional recovery improved, as well as the length of hospital stay, and 30 days postoperative complications were reduced among study group than control group. This could be due to the successful implementation of postoperative enhanced recovery elements for the study groups. Successful implementation of postoperative enhanced recovery elements requires a multidisciplinary team approach. All health care providers worked cohesively to target the goals of enhanced recovery.

However, it is not possible from the present findings to determine which elements of the ERAS resulted in the improved outcome, if it was looked for each element of enhanced recovery. It is an evidence-based practice like early nutrition: in this study, it was started by chewing gum 6 h postoperatively. Postoperative ileus (POI) is an unlikely and common sequel to abdominal surgery, which means a temporary inhibition of normal peristaltic activity of gastrointestinal track, typically lasting for 3 to 4 days after surgery; all portions of gastrointestinal track are involved by POI. The small intestine is the first to regain its functions, usually within the first 24 h, afterward stomach in about 24 to 48 h and usually it takes 48 to 72 h for large intestine to regain its function.

This could be interpreted by the process of chewing gum stimulates digestive system nerves which triggers the release of gastrointestinal hormones and production of both saliva and pancreatic secretions which offers significant benefits in reducing the time to resolution of ileus. The result of the current study reflects this scientific fact as there was a statistically significant difference between the two groups regarding functional recovery. The study group had early first bowel movement, flatus passage, first stool passage and better quality of life during hospitalization period compared to control group. This agrees with a randomized controlled trial by [14] which discovered that gum chewing had a positive effect on improvement of gastrointestinal motility in post abdominal surgery patients.

Oral liquids followed chewing gum. It started early first 24 h postoperative which may also be contributed to early return of gastrointestinal motility. A recent study by [15] support our finding as their results demonstrated that patients who received early oral feeding showed a shorter length of hospital stay, lower pain scores, and shorter postoperative ileus duration than patients in the traditional postoperative care. Also, they noted no duodenal repair site leak in the early oral feeding group.

Nasogastric tube for decompression is another element of enhanced recovery that had been removed after 24 h. postoperative. A meta-analysis study by [16] support this issue as they reported that the use of nasogastric decompression does not always prevent aspiration and may even cause an increased incidence of postoperative complications.

Another element was early ambulation which was also adopted as it reduces the risk of postoperative complications, accelerates the recovery and thereby reducing hospital length of stay. A recent systematic review and meta-analysis by [17] showed that postoperative enhanced recovery for perforated peptic ulcer significantly shortened hospital stay in the studied cohort without increasing the risk of postoperative complications.

Regarding postoperative complications that occurred within 30 days postoperative, the results of the present study illustrated that the number of patients who developed pneumonia, prolonged ileus, and surgical site infection significantly decreased in study group than control group. This difference may be attributed to conducting postoperative enhanced recovery for study group which led to better outcome.

Congruent with these findings, several studies [10, 18–20] support these study findings as they found the application of ERAS protocols with certain modifications in the management of perforated duodenal ulcers yields significant outcomes in terms of early functional recovery for the time to first flatus, first stool passage, and removal of drain. Also, there was a significant reduction in postoperative morbidity rate and shorter LOH in ERAS group compared with standard care group.

Additionally, a meta-analysis of randomized trials involving either ERAS or FTS for abdominal or pelvic surgery by [21] support our finding as they added that ERAS/FTS protocols are powerful tools to prevent HAIs as they found enhanced recovery after surgery (ERAS) and fast track surgery (FTS) protocols are associated with reduction in healthcare-associated infection (HAIs).

After 30 days postoperative the results of the current study demonstrated a significant improvement in all domains of gastrointestinal quality of life index for the study group compared to control group. This could be explained by a healed ulcer improving digestion, absorption of nutrients hence positively affecting on various physiological systems such as immune system, central nervous system and hepato-endocrine system, thus contributing to higher energy, endurance, overall health, wellbeing, and overall quality of life.

## Conclusion

The implementation of postoperative enhanced recovery in the management of perforated peptic ulcers yields significant outcomes in terms of quicker gastrointestinal functional recovery, reduced length of hospital stay, fewer postoperative complications, and better quality of life.

## Data Availability

No datasets were generated or analysed during the current study.

## Abbreviations

DVT: Deep venous thrombosis
ERAS: Enhanced recovery after surgery
FTS: Fast track surgery
GIQLI: Gastrointestinal quality of life index
GIT: Gastrointestinal
*H. pylori*: *Helicobacter pylori*
HAI: Hospital aquired infection
IV: Intravenous
NSAID: Non-steroidal inflammatory drugs
PPU: Perforated peptic ulcer
PUD: Peptic ulcer disease
QOL: Quality of life
